# The Utility of Arterial Spin Labeling MRI in Medial Temporal Lobe as a Vascular Biomarker in Alzheimer’s Disease Spectrum: A Systematic Review and Meta-Analysis

**DOI:** 10.3390/diagnostics12122967

**Published:** 2022-11-27

**Authors:** Efthymia Maria Kapasouri, Diomidis C. Ioannidis, Donnie Cameron, Vassilios S. Vassiliou, Michael Hornberger

**Affiliations:** 1 Cambridge University Hospitals, Hills Road, Cambridge CB2 0QQ, UK; 2 Norwich Medical School, University of East Anglia, Norwich NR4 7UQ, UK; 3 C. J. Gorter MRI Center, Department of Radiology, Leiden University Medical Center, Albinusdreef 2, 2333 ZA Leiden, The Netherlands; 4 Department of Cardiology, Norfolk and Norwich University Hospital, Norwich NR4 7UY, UK

**Keywords:** arterial spin labeling, cerebral blood flow, medial temporal lobe, dementia, mild cognitive decline, ASL, MRI

## Abstract

We sought to systematically review and meta-analy the role of cerebral blood flow (CBF) in the medial temporal lobe (MTL) using arterial spin labeling magnetic resonance imaging (ASL-MRI) and compare this in patients with Alzheimer’s disease (AD), individuals with mild cognitive impairment (MCI), and cognitively normal adults (CN). The prevalence of AD is increasing and leading to high healthcare costs. A potential biomarker that can identify people at risk of developing AD, whilst cognition is normal or only mildly affected, will enable risk-stratification and potential therapeutic interventions in the future. All studies investigated the role of CBF in the MTL and compared this among AD, MCI, and CN participants. A total of 26 studies were included in the systematic review and 11 in the meta-analysis. Three separate meta-analyses were conducted. Four studies compared CBF in the hippocampus of AD compared with the CN group and showed that AD participants had 2.8 mL/min/100 g lower perfusion compared with the CN group. Eight studies compared perfusion in the hippocampus of MCI vs. CN group, which showed no difference. Three studies compared perfusion in the MTL of MCI vs. CN participants and showed no statistically significant differences. CBF measured via ASL-MRI showed impairment in AD compared with the CN group in subregions of the MTL. CBF difference was significant in hippocampus between the AD and CN groups. However, MCI and CN group showed no significant difference in subregions of MTL.

## 1. Introduction

Dementia is a neurodegenerative syndrome that predominantly affects older adults. It affects cognition irreversibly while consciousness remains intact. Cognitive domains, namely episodic memory, language, executive functions, and visual–spatial skills deteriorate progressively [1]. Patients become gradually more dependent on relatives and carers for essential daily-living tasks. As life expectancy is extended, the prevalence rate of dementia increases. According to the World Health Organisation, 50 million people worldwide are living with dementia, and this number is anticipated to reach 152 million by 2050 [2]. In 2019, approximately 885,000 dementia patients were living in the UK and their overall care cost for that year was estimated at GBP 34.7 billion [3]. By 2040, UK patients will have risen to 1.6 million and the cost of their care will have reached GBP 94.1 billion [3]. As no preventive treatment is available, a better understanding of the disease process is needed if we are to decelerate the degenerative process by applying pharmaceutical interventions. To this end, research on potential biomarkers and preclinical diagnosis will underpin the research in treatment.

The neurodegenerative process of AD begins years or even decades before symptomatic disease. According to NIA-AA, it can manifest with various symptoms. These include reduced ability to acquire and maintain new information, impaired management of complex tasks, reduced visuospatial skills, impaired language function, as well as personality changes [4]. AD is considered a continuum in which earlier asymptomatic stages can be detected on a biological level [5]. Towards this direction, the National Institute on Aging and Alzheimer’s Association (NIA-AA) proposed a research framework of diagnostic biomarkers: the AT(N) classification. AT(N) incorporates several imaging and biological tests that act as biomarkers. Individual biomarkers are detected in various stages of the AD continuum and are complementary to the clinical criteria in the diagnosis of AD dementia and its earlier stages [6,7].

Recent research has revealed emerging biomarkers related to the vascular component of AD. According to the vascular hypothesis, neurodegeneration in AD is the outcome of vascular disturbances in the brain [8]. Cerebrovascular impairment plays a key role in AD pathogenesis. Chronic brain hypoperfusion causes oxidative stress and inflammation [9]. Oxidative stress can cause blood–brain barrier impairment which reduces the clearance of beta amyloid. In addition, there is a higher expression of proinflammatory cytokines—IL1β and TNFa—in brain cells of MCI and AD patients, compared with controls. In turn, accumulation of beta amyloid triggers—through the production of IL-6—the differentiation of microglial cells, resulting in further neuroinflammation and blood–brain barrier dysfunction [10]. Badji et al. (2020) suggested the incorporation of biomarkers which reflect vascular deficiencies, such as lacunes, WMH, microbleeds, and cerebral blood flow changes [11]. Identifying groups of people with the aforementioned changes offers a wider window for interventions to delay cognitive impairment.

CBF is considered a potential vascular biomarker (V). CBF can be measured through different imaging modalities [12]. Invasive available techniques are single-photon emission tomography (SPECT), H2(15)O positron emission tomography (PET), Xenon-enhanced CT, and dynamic susceptibility contrast magnetic resonance imaging (DSC-MRI). These modalities use exogenous contrast agents [12]. SPECT and FDG PET have been widely used in the diagnosis of MCI and AD for over 3 decades [13]. SPECT showed a specificity of 80% and a sensitivity of 90% in distinguishing AD patients from cognitively normal controls. For the same purpose, FDG-PET’s sensitivity was 85% and specificity 89% [14]. O’ Brien et al. directly compared the efficacy of the aforementioned modalities, showing statistically significant superiority of FDG PET compared with SPECT [15]. Cost-effectiveness of both SPECT and PET is still an area of debate. There are groups who maintain that early diagnosis with these two modalities is not cost-effective due to the lack of definitive treatment and the low estimation of quality-adjusted years. On the other hand, studies utilizing PET in the US and SPECT in the EU showed that early diagnosis of AD can reduce the annual patient healthcare cost by 15%. This reduction is believed to be greater than the cost of annual testing with these modalities. Reference [13] presents DSC-MRI, a relatively novel technique in current clinical practice. This technique utilizes gadolinium-based agents to measure CBF, cerebrovascular volume (CBV), and mean transit time [16]. DSC-MRI is a functional method, which detected reduced regional CBV in AD patients compared with healthy controls, with a reported sensitivity and specificity of approximately 90% [17].

There is also a non-invasive MRI technique, arterial spin labeling MRI (ASL-MRI). ASL-MRI captures changes in tissue perfusion of the whole brain or specific regions of interest using arterial water molecules as an endogenous tracer [18]. ASL can be easily added to conventional brain MRI, requiring 5 min of additional scan time [19]. Preliminary data indicate that ASL-MRI can be used as a predictor of cognitive impairment, as it can detect vascular changes that structural MRI overlooks. During the last decade, significant efforts to standardise ASL-MRI have been made, although the recommended implementation is not universally applied [20,21]. Studies have shown an overlap of hypometabolism patterns acquired via FDG-PET and hypoperfusion through ASL-MRI between AD patients and differentiation of AD from cognitively normal individuals. Although ASL-MRI has been shown to have a lower sensitivity compared with SPECT and FDG-PET, ASL-MRI is still considered a promising alternative due to its advantages [22,23,24]. Another aspect that should be taken into consideration is that MRI is a more accessible modality in comparison with both PET and SPECT. According to the official stats of Eurostat, availability of MRI ranged among EU countries from 0.5 to 3.4 scanners per 100,000 of population. The same measure for G-cameras—including SPECT—was 0.3–1.8. PET scanners ranged from 0 in Lichtenstein to a maximum of 0.8 per 100,000 of population in Denmark [25].

Perfusion patterns in the precuneus and posterior cingulate gyrus have shown a straightforward correlation to cognitive decline [26,27]. Medial temporal lobe (MTL) is another region of research interest that has already been studied via structural MRI measuring its volume. Atrophy of the MTL is an established biomarker that should not be used in isolation for early diagnosis of AD [28]. CBF in the MTL was also studied and has shown possible correlation between it and the evolution of AD [29,30]. Due to the limited number of publications and their complexity, the potential of perfusion in MTL for assessing AD progression could not be adequately assessed by reviews published in the previous decade [27]. However, recently there has been an increase in the number of studies aiming to evaluate MTL perfusion as a potential biomarker in AD. Many of these studies used ASL-MRI due to its aforementioned advantages. We believe that a systematic analysis of these newly emerging data would provide a comprehensive evaluation of the relationship between MTL perfusion and AD.

This systematic review focuses on the use of ASL-MRI as a potential vascular biomarker (V). Our aim is to evaluate the available data of CBF changes in the medial temporal lobe measured via resting state ASL-MRI in adults with cognitive decline. To accomplish this, we compared perfusion patterns in participants with AD with cognitively normal elderly adults (CN) and MCI to CN.

## 2. Materials and Methods

We followed the PRISMA 2020 statement to conduct this systematic review [31]. The research sources were PubMed, Embase/Ovid, and Cochrane library. The databases were searched from inception to 14 February 2021. The search strategy consisted of the following terms:

(Cognit* OR Alzheimer* OR Memor*) AND (cerebral blood flow OR CBF OR arterial spin labeling OR ASL OR perfusion) AND (magnetic resonance imaging OR MRI)

No limitations were added. The full search strategy is presented in Appendix A.

We included all studies that fulfilled the following criteria:

1. Observational studies (cohort, case-control, or cross-sectional) or baseline results of interventional studies
2. Human studies written in English
3. Studies that recruited cognitively normal adults as control group
4. Patients diagnosed at any stage of sporadic Alzheimer’s disease and/or participants with mild cognitive impairment, validated with at least one cognitive test
5. Participants who underwent arterial spin labeling magnetic resonance imaging in the resting state (rsASL-MRI).

We excluded conference abstracts, case reports, reviews, and letters to the editor. Studies were excluded if participants with cognitive decline were recruited due to organic pathology (artery occlusion, carotid artery stenosis/stiffness, stroke, intracranial artery stenosis, or CADASIL patients). Postoperative and Parkinson’s disease dementia or cognitive decline in diabetes patients were also beyond our research scope. Early-onset and autosomal dominant AD studies and patients diagnosed with other types of dementia were also excluded.

When more than one study included participants from the same cohort/database, we used the article with the largest sample size or the article that provided the most detailed information on CBF values and cognitive assessment, after discussion between two reviewers (EMK, DCI).

**Figure 1:**
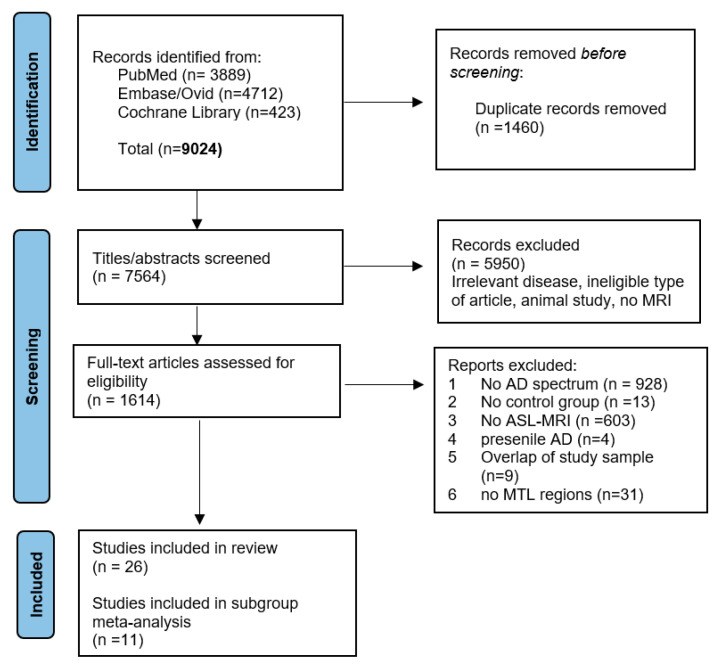
PRISMA flow diagram.

We imported the full reference and the abstracts of the search results in Endnote (Version X9). After de-duplication, two reviewers (EMK, DCI) independently screened the titles and abstracts on the basis of the selection criteria. A third reviewer (VSV) provided adjudication, where necessary. The eligible and inconclusive articles were then assessed on a full-text level using a predesigned algorithm (Appendix B). The selection process is depicted in the Prisma flow diagram recording the reasons of exclusion (Figure 1 in Results section).

The following data were extracted from each eligible study: study details (name of the first author, year of publication, study design), participant characteristics (mean age, sample size, number of female participants, cerebrovascular risk factors, statistical adjustments on age, sex, cerebrovascular risk factors or other parameters, cognitive assessment tests, and the definition of AD or MCI used), MRI data (ASL acquisition type and protocol, MRI specifications, CBF estimation and analysis method, statistical approach, and confounding variables), outcome measurements (mean CBF values (relative or absolute) expressed in millilitres per 100 g per min in a specific region of interest (MTL) and qualitative results of CBF (hypoperfusion/hyperperfusion) in MTL). The data were recorded on predesigned Microsoft Excel (Microsoft 365 version 2202) spreadsheets by two researchers independently.

The Joanna Briggs Institute critical appraisal checklist (JBI) was used to assess the risk of bias of the eligible studies [32]. This tool was the most appropriate for this systematic review, as we included not only cohort and case-control studies but also cross-sectional studies. This checklist detects bias in the design, conduct, and analysis of the studies. Most of the included studies were cross-sectional and the eight domains assessed were as follows:

Questions for the cross-sectional studies are the following:

Q1: Were the criteria for inclusion in the sample clearly defined?

Q2: Were the study subjects and the setting described in detail?

Q3: Was the exposure measured in a valid and reliable way?

Q4: Were objective, standard criteria used for measurement of the condition?

Q5: Were confounding factors identified?

Q6: Were strategies to deal with confounding factors stated?

Q7: Were the outcomes measured in a valid and reliable way?

Q8: Was appropriate statistical analysis used?

The possible answers were: “Yes”, “No”, “Unclear”, or “Not applicable”. Study quality was assessed by two reviewers independently and any disagreement was resolved through discussion. 

A meta-analysis comparing AD vs. CN and MCI vs. CN in relation to CBF flow was also undertaken. Heterogeneity across studies was assessed using the I2 statistic from the standard X2 test. A random-effects model was used in cases of high heterogeneity (I2 > 50%). A sensitivity analysis was also undertaken by excluding each study sequentially. The statistical threshold was set at *p* < 0.05. 

## 3. Results

### 3.1. Search Results

The search selection process is depicted in the PRISMA flow diagram in Figure 1 [31]. We initially identified 9024 references. After de-duplication, 7564 articles were screened at the title/abstract level. We excluded 5950 references because of non-human studies, irrelevant target disease, no MRI exposure, or unclear study design. We screened 1614 references for full-text eligibility. We excluded 1588 studies, as they did not meet the eligibility criteria: no ASL-MRI (n = 603), no AD spectrum (n = 928), no control group (n = 13), presenile AD patients (n = 4), or no MTL regions (n = 31). We also excluded papers due to sample overlap (n = 9). Lastly, we excluded the studies that did not provide CBF results for MTL or its subregions (n = 31).

We assessed the eligibility of several ADNI publications. However, due to high risk of overlap, we decided to include only one study that was more suitable based on the eligibility criteria and studied a large sample of participants.

### 3.2. Included Studies

At the end of the selection process, 26 studies were eligible. We present demographic and participant characteristics in Table 1 [33,34,35,36,37,38,39,40,41,42,43,44,45,46,47,48,49,50,51,52,53,54,55,56,57,58]. Thirteen studies recruited only AD patients, ten studies recruited only MCI participants, and three studies recruited participants from both diagnostic groups. All studies had an observational design and were published after 2000. Sixteen studies were conducted from North America (the US and Canada), seven from Asia, and the remaining three from Europe. APOE4 carriers and cardiovascular risk factors were under-reported, with only three studies including both variables. The sample size of patients diagnosed with AD varied from 12 to 71 participants. The range of MCI participants was also wide, from 9 to 105 volunteers. The mean age of cognitively declined participants ranged from 65 to 84 years. 

For AD diagnosis, the NINCDS-ADRDA, NIA-AA, and revised NINCDS-ADRDA were applied in 11, 2, and 1 studies, respectively [4,59,60]. Most of the studies used the Petersen or Jak criteria for MCI diagnosis [61,62,63].

Imaging characteristics and CBF analysis are depicted in Table 2. A total of 21 out of 26 studies used 3 Tesla MRI scanners, four studies used 1.5 Tesla scanners, while one study was conducted with 4 Tesla scanners. The ASL sequences applied were pulsed ASL (PASL), continuous ASL (CASL), and pseudo-continuous ASL (PCASL) in 9, 5, and 11 studies, respectively. Quantification of CBF was conducted with a wide range of methods, with some studies using more than one approach. A voxel-wise method was applied in 10 out of 26 studies. Regions of interest (ROIs) or other regional approaches were conducted in 20 studies. More than one method was used in five studies. The most frequently used variables as confounders were age and gender (11 out of 26 studies). Partial volume correction was applied in the majority of the studies (20 out of 26).

The main findings of studies that recruited AD patients are summarised in Table 2. Four studies showed decreased perfusion in the MTL for the AD group [34,35,39,40], with two of them reaching statistical significance [34,35]. In contrast, Huang et al. 2018 found higher CBF in the MTL of AD patients without reaching statistical significance [46]. Hypoperfusion was observed in the hippocampus (7 out of 16 studies) [38,44,47,49,50,51]. Four studies showed lower perfusion in the parahippocampal region [35,47,50,51]. Alsop et al. 2008 noted hyperperfusion in hippocampal and parahippocampal regions, which was statistically significant [35]. Lastly, perfusion was decreased in the amygdala in two studies, without reaching statistical significance when comparing AD with CN [48,52].

Moreover, we present the main findings of MCI participants compared with cognitively normal volunteers in Table 2. Two studies showed decreased perfusion in MTL [37,53], but only in one was the difference statistically significant [37]. In addition, higher CBF in the MTL subregions was observed in the Westerberg et al. and Alexopoulos et al. studies, with a significant and a non-significant difference, respectively [33,55]. Furthermore, decreased CBF in the hippocampus was noted in five studies [38,40,44,46,54]. After CBF correction, the difference was non-significant in two of the studies [43,57]. Increased CBF in the hippocampus was described in three studies [33,41,56]. Finally, one study showed a reduction [44], and two an increase of CBF in the parahippocampal region [43,55].

### 3.3. Critical Appraisal

**Figure 2:**
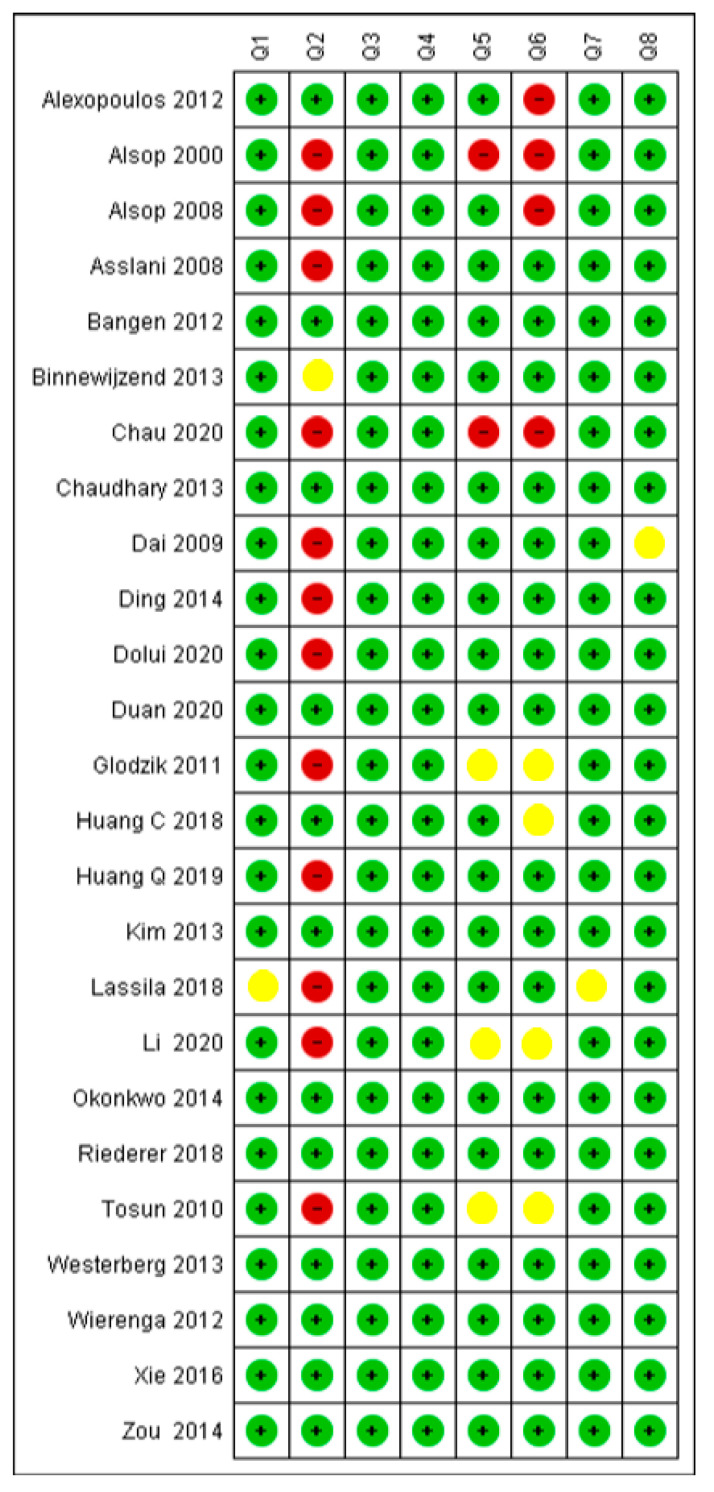
Critical appraisal of included studies (Alexopoulos 2012 [33], Alsop 2000 [34]. Alsop 2008 [35]. Asslani 2008 [36], Bangen 2012 [37], Binnewijzend 2013 [38], Chau 2020 [39], Chaydhary 2013 [40], Dai 2009 [41], Ding 2014 [42], Dolui 2020 [43], Duan 2020 [44], Glodzik 2011 [45], Huang Q 2019 [46], Kim 2013 [47], Lassila 2018 [48], Li 2020 [49], Okonkwo 2014 [50], Riederer 2018 [51], Tosun 2010 [52], Westerberg 2013 [54], Wierenga 2012 [55], Xie 2016 [56] Zou 2014 [57]).

As shown in Figure 2, answers noted in green are “Yes”. Red circles indicate “No”, and yellow was used when the provided information was “unclear”. Eleven studies had a total score of 100% positive answers. Eight studies scored 87.5% and the remaining six studies scored 62.5%. Overall, the risk of bias for the majority of included studies was low. Six studies had a moderate risk of bias as they did not fulfil or provided limited information regarding confounding factors and the study’s setting.

One study (3%) had unclear risk of bias regarding the inclusion criteria of the participants (41). In Question 2 (Q2), we considered a high risk of bias in 11 studies (42%) because the information regarding the setting was limited [34,35,36,39,41,42,43,45,47,49,50,54]. Confounding factors were adjusted for most of the studies (60%). Four studies (15%) did not adjust for confounding factors and were assessed as having a high risk of bias [33,34,35,39]. Another four studies (15%) provided little information regarding the same domain and had an unclear risk of bias [45,46,50,54].

JBI risk of bias quality assessment, green circle indicating that the study addressed well the specific question, red circle that it did not address well the question, and yellow that it provided no information with regard to that question.

### 3.4. Summary of Main Findings and Meta-Analysis

**Figure 3:**
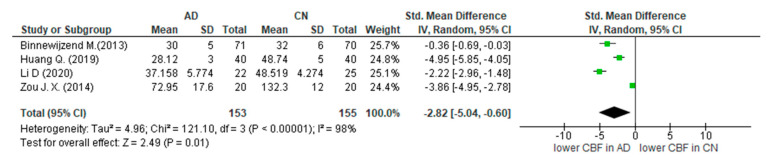
Hippocampus: Alzheimer dementia vs. cognitively normal (Binnewijzend M. (2013) [38], Huang Q. (2019) [47], Li D. (2020) [50], Zou J.X. (2014) [58]).

**Figure 4:**
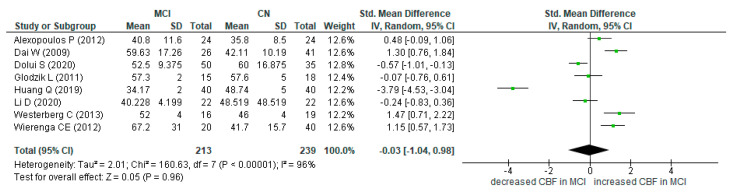
Hippocampus: Mild cognitive impairment vs. cognitively normal (Alexopoulos P. (2012) [33], Dai W. (2009) [41], Dolui S (2020) [43], Glodzik L. (2011) [45], Huang Q. (2019) [47], Li D. (2020) [50], Westerberg C. (2013) [55], Wierenga C.E. (2012) [56]).

**Figure 5:**
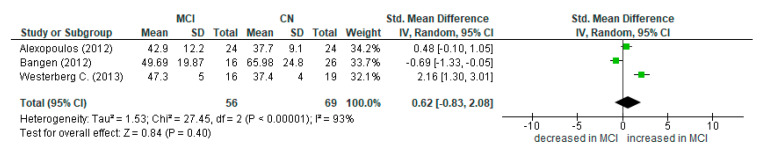
MTL: mild cognitive impairment vs. cognitively normal (Alexopoulos P. (2012) [33], Bangen K.J. (2012) [37], Westerberg C. (2013) [55]).

The volume and the quality of the results on ASL-MRI for detection of MCI participants as well as for AD patients were satisfactory. The results are presented in the summary of findings table (Appendix C). Thirteen studies recruited MCI participants, and 16 studies recruited AD patients. Meta-analysis was difficult to conduct with the inclusion of all studies, as they focused on different subregions of the MTL. Thus, we performed subgroup meta-analyses in the hippocampus for AD-CN groups (Figure 3) as well as in the hippocampus and MTL for MCI-CN groups (Figure 4 and Figure 5). We included these regions in our meta-analysis, as three or more studies provided quantified CBF results, expressed in ml/min/100 g tissue. The CBF values were extracted from text, tables, graphs, or box plots.

This meta-analysis shows that there is significant difference of 2.82 mL/min/100 g between AD and CN in the hippocampus, with AD showing lowed cerebral perfusion. This remained statistically different even following the sensitivity analysis.

There is no statistical difference in cerebral perfusion between MCI and CN.

There is no statistical difference between MCI and CN in MTL perfusion.

## 4. Discussion

### 4.1. Alzheimer’s Disease and ASL

A total of 14 out of 16 studies noted a significantly lower perfusion in the medial temporal lobe of AD patients. These results are in agreement with the hypoperfusion patterns in medial temporal regions depicted in other perfusion imaging modalities, such as PET, SPECT, and DSC [24] and would suggest that hypoperfusion could partly explain the development of AD. Supporting this is a study by Musiek et al. who correlated perfusion patterns using ASL to glucose uptake using fluorodeoxyglucose positron emission tomography (FDG-PET) in AD patients [69]. In their small study, qualitative and quantitative similarities were found, indicating a close relationship between perfusion and metabolic state in different brain regions of AD patients. Adding further evidence for this, a study by Binnewijzend et al. (2016) correlated the lower CBF to later AD stages and a weaker cognitive performance [70]. The number of studies showing statistically significant lower CBF in the MTL of AD patients compared with controls, would demonstrate the important causative relation. CBF can, thus, in the future, become a helpful test in clinical practice in identifying patients at risk. This will be particularly vital if an early disease-modifying therapy becomes available for AD. Of course, CBF assessment is not limited to MRI, and other modalities, such as SPECT and PET, could aid in the earlier diagnosis of AD and, thus, potentially earlier treatment [39].

Of the 16 studies, two showed lack of hypoperfusion in the MTL of AD participants. They specifically showed increase of perfusion in the MTL [46] and the hippocampus [35] but without reaching statistical significance. This transient CBF raise could be explained as the participants recruited had a new onset of AD. Vascular compensatory mechanisms, induced by abnormal neuronal activity, may have still been in place [71]. Inflammatory molecules expression, the disruptions of the blood–brain barrier, the vascular dilation and the high metabolic demands of the atrophic regions could also explain the above [72]. These processes will be eventually exhausted, and low perfusion would be evident in every region of the demented brain.

In conclusion, ASL MRI can detect reduced MTL perfusion in patients at a later stage of AD. However, the results should be interpreted with caution in patients with a new onset of AD, where compensatory mechanisms are still actively influencing CBF measurement. Thus, ASL MRI should complement clinical judgement when managing patients with cognitive decline.

### 4.2. Mild Cognitive Impairment and ASL

The perfusion status of MTL in MCI participants is not as straightforward as in the sample of people with dementia. In three studies, MCI participants had a higher CBF value than the control group. These results were statistically significant in two out of the three studies (66%). In contrast, nine of the included studies presented a statistically significant higher CBF in the control group. It is interesting that in one study, hypoperfusion in the hippocampus was preserved at a significant level after corrections for vascular risk factors and adjusting for partial volume effect between cognitively declined and cognitively normal adults [44]. The wide variability of results on CBF difference between MCI and cognitively normal subjects cannot be overlooked. Although there is no clear explanation for the above, there are certain factors that may influence ASL MRI readings. Cardiovascular risk factors and APOE4 mutation seem to impact CBF readings. Specifically, by recruiting only cognitively normal participants and dividing them into high risk and low risk groups for AD, Fleisher et al. found that the cerebral blood flow in MTL was increased in the high-risk group [73]. In addition, people without dementia who are carriers of the APOE4 allele demonstrated higher CBF in subregions of MTL compared with non-carriers, increasing the possibility of AD conversion in the future [74].

The above hypothesis seems to attract significant interest from the scientific community. Aging, cardiovascular risk factors, APOE mutation, and capillary dysfunction seem to be in the spotlight of recent research. The results of this research show that microcirculation changes due to ageing, hypertension, and genetic risk factors for AD (e.g., the APOE4 allele) are responsible for remodelling of small vessels and, thus, the lowering of oxygen efficacy in the brain [75]. Initially, the brain compensates for the metabolic needs of the neural networks by increasing the CBF of the affected regions. The rise of CBF is sufficient for mild capillary dysfunction. When capillary changes become more evident, the oxygenation demand cannot be compensated for with higher perfusion rates [76]. At this stage, cognitive decline symptoms are pronounced, and CBF is decreased. Recent work confirms this hypothesis by comparing the CBF in MTL of cognitive normal adults, people with objective subtle cognitive decline, and MCI participants [77]. Objective cognitive is defined by Thomas et al. as “performance lesser than 1 standard deviation (SD) below the age, education, and sex-adjusted mean on: (1) two neuropsychological total scores in two different cognitive domains, (2) one neuropsychological total score and one process score, or (3) two process scores”. The group with the objective cognitive decline showed higher CBF in hippocampus than MCI as well as higher CBF than the cognitively normal group, which is suggestive of mild capillary dysfunction.

The above theory could provide a valid explanation of the CBF variation between

MCI and cognitively normal participants in different studies. In addition, high heterogeneity of the included studies could be another reason for the results, and we should always consider the characteristics of the MCI participants (demographics and stage of the disease at the recruitment) and the variety of disease presentation within and between subjects [71]. However, no matter how reasonable the explanations might be, variability in results is a factor that remains present and should be taken into consideration when interpreting ASL MRI readings in MCI patient.

### 4.3. Limitations

We should acknowledge the limitations of our systematic review. We focused our review on English-written studies, implementing a restriction to available included studies. Another weakness is that all but one eligible study were cross-sectional. In this type of study, the cause–outcome/causal relationship cannot be identified, as the disease and the exposure variables are studied simultaneously. The variability of the acquisition protocols and the imaging analysis performed were considerable. These differences in imaging protocols should be considered when we interpret the results of our review. Included studies assessed various comorbidities, namely HTN, stroke, heart disease, and diabetes; however, prediabetes and variable glycemic control were not assessed. A meta-analysis was also conducted. This had significant heterogeneity, but a random-effects model was utilised to account for this. Lastly, the sample size of two studies was less than 30 participants. A very small sample size could bias the outcome, as there is a possibility of our assumptions being confirmed by chance [78].

### 4.4. Recommendations for Future Studies

Despite the limitations, this work confidently addresses whether “CBF in the medial temporal lobe is different between cognitively normal and cognitively declined participants”. This is based on the results of the included studies and without applicability concerns regarding the studied population, imaging test, or cognitive status. This is because cross-sectional studies can demonstrate differences between the studied groups, but the causal relationship remains unclear. For this reason, longitudinal studies are essential to evaluate the effectiveness of the imaging test and to assess the potential role of the CBF changes as an additional biomarker of differentiation between AD and cognitively normal adults.

The potential benefit of incorporating ASL-MRI in general practice should also be assessed. When an MRI is indicated in clinical practice, adding an ASL sequence can be beneficial and of a low additional cost. On the other hand, the plethora of choices in ASL parameters can be a drawback. Multisite comparisons are difficult and expensive to perform. It would be beneficial if the medical community could come together and, through guidelines, mutually agree on a specific protocol so that comparisons of acquired results can be performed directly. Towards this vision, the European working group “ASL in Dementia” and the equivalent USA Perfusion Study group—International Society for Magnetic Resonance in Medicine—have provided indicative acquisition parameters for ASL to standardise the method [21]. The implementation of this consensus could simplify the use of ASL in clinical practice and encourage the adoption of the method in more settings.

ASL-MRI is a perfusion technique that enables the non-invasive quantification of cerebral blood flow. Quantified results could be more objective than descriptive results when researchers define thresholds or a range of values to discriminate cognitive decline from cognitively normal adults [21]. To accomplish this, the brain atrophy and partial volume effect (PVE) should be taken into consideration. PVE correction is essential when CBF values are extracted. From the included studies, only four studies adjusted their results according to PVE [43,53,54,57]. Researchers should account for PVE as a confounder in the design of future studies [20]. PVE-corrected and PVE-uncorrected results can be extremely different, and comparisons among participants of different studies could be a challenge.

### 4.5. Role in Clinical Practice

Arterial spin labeling is a perfusion MRI modality that is relatively inexpensive and easy to perform. Cohort studies and diagnostic test accuracy studies are mandatory to set a common language among clinicians when they interpret ASL results. If these steps are accomplished, ASL-MRI could be considered as an additional test—part of the existing MRI acquisition protocol—to detect people at risk for AD with greater precision and hopefully at an earlier stage. This can be particularly helpful if a therapeutic intervention would become available, whereby early initiation is likely to offer improved outcomes. Authors should discuss the results and how they can be interpreted from the perspective of previous studies and of the working hypotheses. The findings and their implications should be discussed in the broadest context possible. Future research directions may also be highlighted.

## 5. Conclusions

In this comprehensive systematic review and meta-analysis, we show that there is significant difference in the cerebral blood flow of the hippocampus between AD and CN groups. There was no difference in either the cerebral perfusion in the hippocampus or MTL between MCI and CN participants. This work suggests that incorporating cerebral perfusion in routine MRI brain imaging could be beneficial for identifying patients at earlier stages of AD.

## Figures and Tables

**Table 1:** Participant demographics and clinical information.

Study ID	Participants (n)			Female (n)			Mean Age (SD)			MMSE Mean (SD)			Education (Years)			APOE4 Carriers			CVR	Diagnostic Criteria
	AD	MCI	CN	AD	MCI	CN	AD	MCI	CN	AD	MCI	CN	AD	MCI	CN	AD	MCI	CN		
Alexopoulos P (2012) [33] Germany	-	24	24	-	34%	67%	-	69.6 (8.2)	69.6 (8.2)	-	NG	NG	-	NG	NG	-	NG	NG	NG	International working group 2014 [64]
Alsop DC (2000) USA [34]	18	-	11	34%	-	46%	72.2 (6.8)	-	68.9 (7.2)	20.8 (7)	-		NG	-	NG	NG	-	NG	NG	NINCDS-ADRDA
Alsop DC (2008) USA [35]	22	-	16	55%	-	57%	75.6 (9.2)	-	72.6 (8.9)	22.2 (5.9)	-	27.9 (2.6)	15.5 (3.2)	-	14.4 (3.8)	NG	-	NG	NG	NINCDS-ADRDA
Asslani (2008) USA [36]	12	-	20	42%	-	60%	70.7 (8.7)	-	72.1 (6.5)	NG	-	NG	14.5 (3.8)	-	15.8 (2.3)	NG	-	NG	NG	NINCDS-ADRDA
Bangen KJ (2012) USA [37]	-	16	26	-	38%	73%	-	76.88 (7.31)	74.79 (7.98)	-	NG	NG	-	15.56 (2.53)	15.86 (2.33)	-	50%	54%	FSRP, 10-year stroke risk	Jak AJ et al. 2009 [63]
Binnewijzend MAA (2013) Netherlands [38]	71	-	70	55%	-	39%	65 (7)	-	60 (9)	20 (4.6)	-	28 (1.7)	NG	-	NG	NG	-	NG	NG	NINCDS-ADRDA
Chau ACM (2020) Hong Kong [39]	17	-	15	71%	-	80%	75.1 (8.2)	-	71.8 (6.1)	NG	-	NG	NG	-	NG	NG	-	NG	DM, HTN, hyperlipidaemia	NIA-AA
Chaudhary S (2013) Canada [40]	25	-	20	80%	-	55%	72.5 (0.9)	-	71.8 (1.8)	25.7 (1.6)	-	28.4 (0.8)	15.1 (3.4)	-	15.7 (1.2)				NG	NINCDS-ADRDA
Dai W (2009) USA [41]	-	26	41	-	58%	66%	-	83.6 (3.6)	82.1 (3.6)	-	NG	NG	-	NG in years	NG in years	-	23%	12%	HTN, DM, Heart Disease	Cardiovascular Health study criteria
Ding B (2014) China [42]	24	-	21	80%	-	62%	74.58 (6.68)	-	69.64 (5.88)	16 (3.9)	-	29.4 (1)	11.6 (4.2)	-	12.1 (3.4)	NG	-	NG	NG	NINCDS-ADRDA
Dolui S (2020) USA [43]	-	50	35	-	32%	58%	-	70.2 (6.9)	73 (7)	-	27 (6)	30 (5)	-	17.5 (13)	18 (11)	NG	-	NG	NG	Petersen 2004 [61]
Duan (2020) USA [44]	40	-	58	70%	-	55%	84.1 (3.5)	-	83.4 (3.7)	NG	-	NG	13.3 (2.9)	-	14.6 (2.8)	NG	-	NG	HTN, DM, Heart Disease	Cardiovascular Health study criteria
Glodzik L (2011) USA [45]	15	-	18	60%	-	56%	74.9 (8.1)	-	69.9 (6.7)	27.5 (2.4)	-	29.2 (1)	NG	-	NG	NG	-	NG	FSRP	Petersen 2004 [61]
Huang CW (2018) Taiwan [46]	50	-	30	66%	-	60%	73.32 (8.4)	-	71.03 (8.05)	16.78 (5.1)	-	27.07 (1.9)	5.3 (4.51)	-	8.5 (5.22)	54%	-	20%	DM, HTN	Dubois 2010 [65]
Huang Q (2019) China [47]	40	40	40	43%	40%	45%	70.1 (5.7)	68.5 (6.1)	69.1 (5.8)	NG	NG	NG	NG in years	NG in years	NG in years	NG	NG	NG	NG	NIA-AA
Kim SM (2013) South Korea [48]	25	-	25	84%	-	64%	70.9 (9.8)	-	68.4 (5.6)	15.76 (4.39)	-	27.32 (2.8)	NG	-	NG	56%	-	20%	NG	NINCDS-ADRDA
Lassila T (2018) UK [49]	-	9	15	-	67%	54%	-	74.8 (7.8)	73.7 (5.1)	-	NG	NG	-	9.2 (3.4)	11.9 (2.9)	-	NG	NG	NG	NG
Li D (2020) China [50]	22	22	25	59%	55%	60%	71.5 (8.4)	71.8 (8.2)	69.3 (5.2)	18.9 (3.4)	23 (2.7)	29.7 (1.2)	NG	NG	NG	NG	NG	NG	NG	NIA-AA Petersen 2018 [66]
Okonkwo OC (2014) USA [51]	28	23	24	43%	30%	50%	75.09 (9.81)	73.35 (6.95)	75.07 (6.30)	22.04 (3.65)	26.96 (2.01)	29.04 (1.02)	14.57 (3.05)	16.83 (2.95)	16.5 (3.32)	68%	56%	38%	NG	NINCDS-ADRDA, Petersen 2001 [67]
Riederer I (2018) Germany [52]	45	-	11	56%	-	55%	69 (9)	-	65 (8)	22 (4)	-	28.5 (1.1)	12.6 (3.8)	-	12.4 (3)	NG	-	NG	NG	ICD-10, NINCDS-ADRDA
Sanchez DL (2020) USA [53]	-	105	61	-	53%	73%	-	71.01 (7.1)	71.62 (6.44)	-		)	-	16.69 (2.7)	16.38 (2.45)	-	55%	45%	NG	ADNI criteria [68]
Tosun D (2010) USA [54]	24	-	38	38%	-	56%	66.29 (9.99)	-	65.7 (8.25)	21.76 (5.8)	-	29.44 (0.86)	NG	-	NG	NG	-	NG	NG	NINCDS-ADRDA
Westerberg C (2013) USA [55]	-	20	20	-	70%	75%	-	73.6 (NG)	74.6 (NG)	-	27.6 (NG)	29.1 (NG)	-	NG	NG	-	NG	NG	NG	Petersen 2004 [61]
Wierenga CE (2012) USA [56]	-	20	40	-	50%	68%	-	74.8 (11.4)	73.5 (6.8)	-	NG	NG	-	14.5 (2.7)	16.3 (1.8)	-	45%	33%13	NG	Jak AJ et al. 2009 [63]
Xie L (2016) USA [57]		65	62	-	37%	63%	-	74 (6.2)	70.5 (8.8)	-	27.4 (1.7)	29.2 (1)	-	15.8 (3)	16.6 (2.7)	-	NG	NG	NG	Petersen 2004 [61]
Zou JX (2014) China [58]	20	-	20	60%	-	55%	64.84 (8.82)	-	64.94 (7.93)	16.21 (4.01)	-	27.35 (1.01)	10.14 (3.24)	-	11.05 (4.47)	NG	-	NG	NG	NINCDS-ADRDA

AD: Alzheimer’s Disease, MCI: Mild Cognitive Impairment, CN: Cognitive Normal, MMSE: Mini-Mental State Examination, NG: not given, FSRP: Framingham Stroke Risk Profile, NINCDS-ADRDA: National Institute of Neurological and Communicative Disorders and Stroke and the Alzheimer’s Disease and Related Disorders Association, NIA-AA: National Institute on Aging and Alzheimer’s Association, DM: Diabetes mellitus, HTN: hypertension, and ADNI: Alzheimer’s Disease Neuroimaging Initiative.

**Table 2:** Imaging characteristics and CBF analysis.

Study ID	MRI Scan Strength (Tesla)	ASL Sequence	CBF Estimation Method	Partial Volume Correction	Perfusion Change	Regions Studied	
						AD	MCI
Alexopoulos P (2012) Germany [33]	3.0 T	PULSAR	voxel-wise, ROIs	yes	↑	-	MTL and hippocampus, parahippocampal region
Alsop DC (2000) USA [34]	1.5 T	3D ASL	imaged-based, region-based	no	↓ *	MTL	-
Alsop DC (2008) USA [35]	3.0 T	3D CASL	voxel-wise, region-based	yes	↑	Hippocampus, parahippocampal region	-
Asslani (2008) USA [36]	1.5 T	CASL	voxel-wise, ROIs	yes	↓	Right parahippocampal region	-
Bangen KJ (2012) USA [37]	3.0 T	2D PASL	ROIs	yes	↓ *	-	Bilateral and right MTL
Binnewijzend MAA (2013) Netherlands [38]	3.0 T	3D PCASL	ROIs	yes	↓ *	Hippocampus. Results adjusted for age, sex, and WMH severity.	-
Chau ACM (2020) Hong Kong [39]	3.0 T	2D PCASL	ROIs	yes	↓	MTL Adjusted for age, gender, and GM volume.	-
Chaudhary S (2013) Canada [40]	3.0 T	3D PCASL	ROIs	yes	↓	MTL	-
Dai W (2009) USA [41]	3.0 T	CASL	ROIs	yes	↑ *	-	Right amygdala and left hippocampus. Results adjusted for age, sex and hypertension history.
Ding B (2014) China [42]	3.0 T	PCASL	voxel-wise	no	↓ *	Left limbic lobe and parahippocampal region	-
Dolui S (2020) USA [43]	3.0 T	2D PCASL	voxel-wise, ROIs	yes	↓		Hippocampus
Duan (2020) USA [44]	1.5 T	CASL	voxel-wise	yes	↓ *	Left hippocampus	-
Glodzik L (2011) USA [45]	3.0 T	PASL	ROIs	yes	↔	Right hippocampus	-
Huang CW (2018) Taiwan [46]	1.5 T	PCASL	voxel-wise	yes	↑	MTL	-
Huang Q (2019) China [47]	3.0 T	3D PCASL	ROIs	no	↓ *	Hippocampus	Hippocampus
Kim SM (2013) South Korea [48]	3.0 T	PASL	voxel-wise	yes	↓	Left and right parahippocampal regions as well as left and right amygdala. Results adjusted for APOE status.	-
Lassila T (2018) UK [49]	3.0 T	PCASL	z-scores	no	↓ *	-	Left hippocampus
Li D (2020) China [50]	3.0 T	3D PCASL	ROIs	no	↓ *	Hippocampus	Hippocampus
Okonkwo OC (2014) USA [51]	3.0 T	PCASL	voxel-wise	yes	↓	Left parahippocampal region	Left parahippocampal region
Riederer I (2018) Germany [52]	3.0 T	PASL	voxel-wise	yes	↓	Hippocampus, parahippocampal region, amygdala	-
Sanchez DL (2020) USA [53]	3.0 T	PASL	ROIs	yes	↓	-	MTL decreased over 3 years
Tosun D (2010) USA [54]	4.0 T	CASL	ROIs	yes	↓	Left and right hippoocampus	-
Westerberg C (2013) USA [55]	3.0 T	2D PASL	ROIs	yes	↑ *	-	Parahippocampal and entorhinal regions
Wierenga CE (2012) USA [56]	3.0 T	PASL	voxel-wise, ROIs	yes	↑ *	-	Right hippocampus
Xie L (2016) USA [57]	3.0 T	2D PCASL	ROIs	yes	↓ *	-	Left hippocampus No significance remained after correction for multiple comparisons
Zou JX (2014) China [58]	3.0 T	3D PASL	ROIs	no	↓ *	Hippocampus, bilaterally	-

↑: increase ↓: decrease, ↔: no change, * statistically significant, -: not applicable/not studied. Comparisons made between AD and CN groups as well as MCI and CN groups.

**Figure A1:**
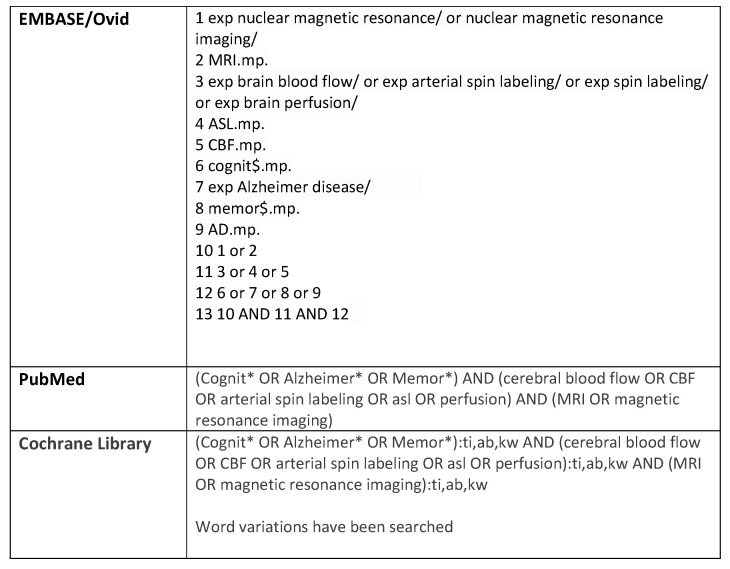
Full search strategy.

**Figure A2:**
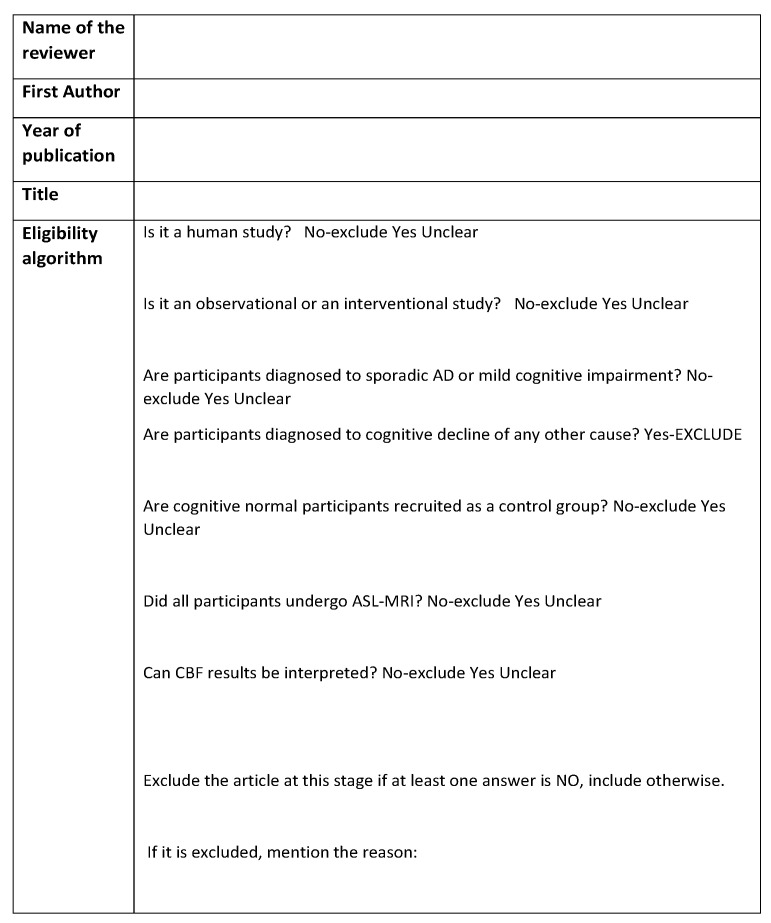
Eligibility template.

## Data Availability

All the available data are included in the manuscript.

## Appendix C

**Table A1:** Summary of findings.

Differences in CBF of MTL in people with cognitive decline compared with cognitively normal adults	
Patient population	People with cognitive decline
Alzheimer’s disease patients	included studies (n = 16)
AD criteria	NINCDS-ADRDA (n = 10) revised NINCDS-ADRDA (n = 2) ICD-10 and/or NINCDS-ADRDA (n = 1) NIA-AA (n = 3)
Mild cognitive impairment	included studies (n = 12)
MCI criteria	Petersen criteria (n = 4) Petersen and modified Petersen criteria (n = 2) modified Petersen criteria (n = 3) Reisberg 1993 (n = 1) ADNI criteria (n = 1) unknown (n = 1)
Imaging test	Arterial spin labelling (perfusion MRI)
MRI strength	3 T (n = 21) 1.5 T (n = 4) 4 T (n = 1)
ASL sequence	PCASL (n = 11) CASL (n = 5) PASL (n = 8) unclear/other (n = 2)
Readout	2D-EPI (n = 5) 3D (n = 7) unclear (n = 15)
Comparator	Cognitively normal adults
Outcome	Cerebral blood flow in MTL
Alzheimer’s disease patients (CBF analysis)	voxel-wise (n = 6) ROIs (n = 8) other methods (n = 2)
Mild cognitive impairment (CBF analysis)	voxel-wise (n = 3) ROIs (n = 10) other methods (n = 2)
Included studies	Observational studies (cross-sectional n = 25, cohort n = 1)
Quality concerns	Patients’ characteristics were not always adequately described and confounding factors were moderately reported. Concerns regarding reproducibility of the investigated method were generally low.
Limitations	Lack of standard methodology in ASL-MRI process. Variety of regions of interest (ROIs) among studies
Conclusions	There is need for conducting longitudinal studies with a standardised methodological protocol of ASL-MRI with larger population samples.

## References

[1] Elahi F.M., Miller B.L. (2017). A clinicopathological approach to the diagnosis of dementia. Nat. Rev. Neurol..

[2] World Health Organization Dementia. https://www.who.int/news-room/fact-sheets/detail/dementia.

[3] Wittenberg R., Hu B., Barraza-Araiza L., Rehill A. (2019). Projections of Older People Living with Dementia and Costs of Dementia Care in the United Kingdom, 2019–2040.

[4] McKhann G.M., Knopman D.S., Chertkow H., Hyman B.T., Jack C.R., Kawas C.H., Klunk W.E., Koroshetz W.J., Manly J.J., Mayeux R. (2011). The diagnosis of dementia due to Alzheimer’s disease: Recommendations from the National Institute on Aging-Alzheimer’s Association workgroups on diagnostic guidelines for Alzheimer’s disease. Alzheimer’s Dement..

[5] Vermunt L., Sikkes S.A.M., van den Hout A., Handels R., Bos I., van der Flier W.M., Kern S., Ousset P.J., Maruff P., Skoog I. (2019). Duration of preclinical, prodromal, and dementia stages of Alzheimer’s disease in relation to age, sex, and APOE genotype. Alzheimer’s Dement..

[6] Lloret A., Esteve D., Lloret M.A., Cervera-Ferri A., Lopez B., Nepomuceno M., Monllor P. (2019). When Does Alzheimer’s Disease Really Start? The Role of Biomarkers. Int. J. Mol. Sci..

[7] Dubois B., Villain N., Frisoni G.B., Rabinovici G.D., Sabbagh M., Cappa S., Bejanin A., Bombois S., Epelbaum S., Teichmann M. (2021). Clinical diagnosis of Alzheimer’s disease: Recommendations of the International Working Group. Lancet Neurol..

[8] de la Torre J. (2018). The Vascular Hypothesis of Alzheimer’s Disease: A Key to Preclinical Prediction of Dementia Using Neuroimaging. J. Alzheimer’s Dis..

[9] Scheffer S., Hermkens D.M.A., van der Weerd L., de Vries H.E., Daemen M.J.A.P. (2021). Vascular Hypothesis of Alzheimer Disease: Topical Review of Mouse Models. Arterioscler. Thromb. Vasc. Biol..

[10] Kinney J.W., Bemiller S.M., Murtishaw A.S., Leisgang A.M., Salazar A.M., Lamb B.T. (2018). Inflammation as a central mechanism in Alzheimer’s disease. Alzheimer’s Dement. Transl. Res. Clin. Interv..

[11] Badji A., Westman E. (2020). Cerebrovascular pathology in Alzheimer’s disease: Hopes and gaps. Psychiatry Res. Neuroimaging.

[12] Zeng H.M., Han H.B., Zhang Q.F., Bai H. (2021). Application of modern neuroimaging technology in the diagnosis and study of Alzheimer’s disease. Neural Regen. Res..

[13] Ferrando R., Damian A. (2021). Brain SPECT as a Biomarker of Neurodegeneration in Dementia in the Era of Molecular Imaging: Still a Valid Option?. Front. Neurol..

[14] Valotassiou V., Malamitsi J., Papatriantafyllou J., Dardiotis E., Tsougos I., Psimadas D., Alexiou S., Hadjigeorgiou G., Georgoulias P. (2018). SPECT and PET imaging in Alzheimer’s disease. Ann. Nucl. Med..

[15] O’Brien J.T., Firbank M.J., Davison C., Barnett N., Bamford C., Donaldson C., Olsen K., Herholz K., Williams D., Lloyd J. (2014). 18F-FDG PET and perfusion SPECT in the diagnosis of Alzheimer and Lewy body dementias. J. Nucl. Med..

[16] Quarles C.C., Bell L.C., Stokes A.M. (2019). Imaging vascular and hemodynamic features of the brain using dynamic susceptibility contrast and dynamic contrast enhanced MRI. Neuroimage.

[17] Bozzao A., Floris R., Baviera M.E., Apruzzese A., Simonetti G. (2001). Diffusion and perfusion MR imaging in cases of Alzheimer’s disease: Correlations with cortical atrophy and lesion load. AJNR Am. J. Neuroradiol..

[18] Eurostat Availability of Technical Resources in Hospitals. https://ec.europa.eu/eurostat/statisticsexplained/index.php?title=Healthcare_resource_statistics_-_technical_resources_and_medical_technology&oldid=575172#Use_of_medical_technology.

[19] Williams D.S., Detre J.A., Leigh J.S., Koretsky A.P. (1992). Magnetic resonance imaging of perfusion using spin inversion of arterial water. Proc. Natl. Acad. Sci. USA.

[20] Grade M., Hernandez-Tamames J.A., Pizzini F.B., Achten E., Golay X., Smits M. (2015). A neuroradiologist’s guide to arterial spin labeling MRI in clinical practice. Neuroradiology.

[21] Alsop D.C., Detre J.A., Golay X., Günther M., Hendrikse J., Hernandez-Garcia L., Lu H., MacIntosh B.J., Parkes L.M., Smits M. (2015). Recommended implementation of arterial spin-labeled perfusion MRI for clinical applications: A consensus of the ISMRM perfusion study group and the European consortium for ASL in dementia. Magn. Reson. Med..

[22] Morinaga A., Ono K., Ikeda T., Ikeda Y., Shima K., Noguchi-Shinohara M., Samuraki M., Yanase D., Yoshita M., Iwasa K. (2010). A comparison of the diagnostic sensitivity of MRI, CBF-SPECT, FDG-PET and cerebrospinal fluid biomarkers for detecting Alzheimer’s disease in a memory clinic. Dement. Geriatr. Cogn. Disord..

[23] Fällmar D., Haller S., Lilja J., Danfors T., Kilander L., Tolboom N., Egger K., Kellner E., Croon P.M., Verfaillie S.C. (2017). Arterial spin labeling-based Z-maps have high specificity and positive predictive value for neurodegenerative dementia compared to FDG-PET. Eur. Radiol..

[24] Chen Y., Wolk D.A., Reddin J.S., Korczykowski M., Martinez P.M., Musiek E.S., Newberg A.B., Julin P., Arnold S.E., Greenberg J.H. (2011). Voxel-level comparison of arterial spin-labeled perfusion MRI and FDG-PET in Alzheimer disease. Neurology.

[25] Verfaillie S.C., Adriaanse S.M., Binnewijzend M.A., Benedictus M.R., Ossenkoppele R., Wattjes M.P., Pijnenburg Y.A., van der Flier W.M., Lammertsma A.A., Kuijer J. (2015). Cerebral perfusion and glucose metabolism in Alzheimer’s disease and frontotemporal dementia: Two sides of the same coin?. Eur. Radiol..

[26] Wang Z. (2014). Characterizing early Alzheimer’s disease and disease progression using hippocampal volume and arterial spin labeling perfusion MRI. J. Alzheimers Dis..

[27] Alsop D.C., Dai W., Grossman M., Detre J.A. (2010). Arterial spin labeling blood flow MRI: Its role in the early characterization of Alzheimer’s disease. J. Alzheimers Dis..

[28] Lombardi G., Crescioli G., Cavedo E., Lucenteforte E., Casazza G., Bellatorre A.G., Lista C., Costantino G., Frisoni G., Virgili G. (2020). Structural magnetic resonance imaging for the early diagnosis of dementia due to Alzheimer’s disease in people with mild cognitive impairment. Cochrane Database Syst. Rev..

[29] Caroli A., Testa C., Geroldi C., Nobili F., Guerra U.P., Bonetti M., Frisoni G.B. (2007). Brain perfusion correlates of medial temporal lobe atrophy and white matter hyperintensities in mild cognitive impairment. J. Neurol..

[30] Lacalle-Aurioles M., Mateos-Pérez J.M., Guzmán-De-Villoria J.A., Olazarán J., Cruz-Orduña I., Alemán-Gómez Y., Martino M.E., Desco M. (2014). Cerebral blood flow is an earlier indicator of perfusion abnormalities than cerebral blood volume in Alzheimer’s disease. J. Cereb. Blood Flow Metab..

[31] Page M.J., McKenzie J.E., Bossuyt P.M., Boutron I., Hoffmann T.C., Mulrow C.D., Shamseer L., Tetzlaff J.M., Akl E.A., Brennan S.E. (2021). The PRISMA 2020 statement: An updated guideline for reporting systematic reviews. Syst. Rev..

[32] Zeng X., Zhang Y., Kwong J.S., Zhang C., Li S., Sun F., Niu Y., Du L. (2015). The methodological quality assessment tools for preclinical and clinical studies, systematic review and meta-analysis, and clinical practice guideline: A systematic review. J. Evid. Based Med..

[33] Alexopoulos P., Sorg C., Förschler A., Grimmer T., Skokou M., Wohlschläger A., Perneczky R., Zimmer C., Kurz A., Preibisch C. (2012). Perfusion abnormalities in mild cognitive impairment and mild dementia in Alzheimer’s disease measured by pulsed arterial spin labeling MRI. Eur. Arch. Psychiatry Clin. Neurosci..

[34] Alsop D.C., Detre J.A., Grossman M. (2000). Assessment of cerebral blood flow in Alzheimer’s disease by spin-labeled magnetic resonance imaging. Ann. Neurol..

[35] Alsop D.C., Casement M., de Bazelaire C., Fong T., Press D.Z. (2008). Hippocampal hyperperfusion in Alzheimer’s disease. Neuroimage.

[36] Asllani I., Habeck C., Scarmeas N., Borogovac A., Brown T.R., Stern Y. (2008). Multivariate and univariate analysis of continuous arterial spin labeling perfusion MRI in Alzheimer’s disease. J. Cereb. Blood Flow Metab..

[37] Bangen K.J., Restom K., Liu T.T., Wierenga C.E., Jak A.J., Salmon D.P., Bondi M.W. (2012). Assessment of Alzheimer’s disease risk with functional magnetic resonance imaging: An arterial spin labeling study. J. Alzheimers Dis..

[38] Binnewijzend M.A., Kuijer J.P., Benedictus M.R., van der Flier W.M., Wink A.M., Wattjes M.P., van Berckel B.N., Scheltens P., Barkhof F. (2013). Cerebral blood flow measured with 3D pseudocontinuous arterial spin-labeling MR imaging in Alzheimer disease and mild cognitive impairment: A marker for disease severity. Radiology.

[39] Chau A.C., Cheung E.Y., Chan K.H., Chow W.S., Shea Y.F., Chiu P.K., Mak H.K. (2020). Impaired cerebral blood flow in type 2 diabetes mellitus—A comparative study with subjective cognitive decline, vascular dementia and Alzheimer’s disease subjects. Neuroimage Clin..

[40] Chaudhary S., Scouten A., Schwindt G., Janik R., Lee W., Sled J.G., Black S.E., Stefanovic B. (2013). Hemodynamic effects of cholinesterase inhibition in mild Alzheimer’s disease. J. Magn. Reason. Imaging.

[41] Dai W., Lopez O.L., Carmichael O.T., Becker J.T., Kuller L.H., Gach H.M. (2009). Mild cognitive impairment and alzheimer disease: Patterns of altered cerebral blood flow at MR imaging. Radiology.

[42] Ding B., Ling H.W., Zhang Y., Huang J., Zhang H., Wang T., Yan F.H. (2014). Pattern of cerebral hyperperfusion in Alzheimer’s disease and amnestic mild cognitive impairment using voxel-based analysis of 3D arterial spin-labeling imaging: Initial experience. Clin. Interv. Aging.

[43] Dolui S., Li Z., Nasrallah I.M., Detre J.A., Wolk D.A. (2020). Arterial spin labeling versus 18F-FDG-PET to identify mild cognitive impairment. Neuroimage Clin..

[44] Duan W., Sehrawat P., Balachandrasekaran A., Bhumkar A.B., Boraste P.B., Becker J.T., Kuller L.H., Lopez O.L., Gach H.M., Dai W. (2020). Cerebral Blood Flow Is Associated with Diagnostic Class and Cognitive Decline in Alzheimer’s Disease. J. Alzheimers Dis..

[45] Glodzik L., Rusinek H., Brys M., Tsui W.H., Switalski R., Mosconi L., Mistur R., Pirraglia E., De Santi S., Li Y. (2011). Framingham cardiovascular risk profile correlates with impaired hippocampal and cortical vasoreactivity to hypercapnia. J. Cereb. Blood Flow Metab..

[46] Huang C.W., Hsu S.W., Chang Y.T., Huang S.H., Huang Y.C., Lee C.C., Chang W.N., Lui C.C., Chen N.C., Chang C.C. (2018). Cerebral Perfusion Insufficiency and Relationships with Cognitive Deficits in Alzheimer’s Disease: A Multiparametric Neuroimaging Study. Sci. Rep..

[47] Huang Q., Cao X., Chai X., Wang X., Xu L., Xiao C. (2019). Three-dimensional pseudocontinuous arterial spin labeling and susceptibility-weighted imaging associated with clinical progression in amnestic mild cognitive impairment and Alzheimer’s disease. Medicine.

[48] Kim S.M., Kim M.J., Rhee H.Y., Ryu C.W., Kim E.J., Petersen E.T., Jahng G.H. (2013). Regional cerebral perfusion in patients with Alzheimer’s disease and mild cognitive impairment: Effect of APOE epsilon4 allele. Neuroradiology.

[49] Lassila T., Di Marco L.Y., Mitolo M., Iaia V., Levedianos G., Venneri A., Frangi A.F. (2018). Screening for Cognitive Impairment by Model-Assisted Cerebral Blood Flow Estimation. IEEE Trans. Biomed. Eng..

[50] Li D., Liu Y., Zeng X., Xiong Z., Yao Y., Liang D., Qu H., Xiang H., Yang Z., Nie L. (2020). Quantitative Study of the Changes in Cerebral Blood Flow and Iron Deposition During Progression of Alzheimer’s Disease. J. Alzheimers Dis..

[51] Okonkwo O.C., Xu G., Oh J.M., Dowling N.M., Carlsson C.M., Gallagher C.L., Birdsill A.C., Palotti M., Wharton W., Hermann B.P. (2014). Cerebral blood flow is diminished in asymptomatic middle-aged adults with maternal history of Alzheimer’s disease. Cereb. Cortex.

[52] Riederer I., Bohn K.P., Preibisch C., Wiedemann E., Zimmer C., Alexopoulos P., Förster S. (2018). Alzheimer Disease and Mild Cognitive Impairment: Integrated Pulsed Arterial Spin-Labeling MRI and 18F-FDG PET. Radiology.

[53] Sanchez D.L., Thomas K.R., Edmonds E.C., Bondi M.W., Bangen K.J. (2020). Alzheimer’s Disease Neuroimaging Initiative. Regional Hypoperfusion Predicts Decline in Everyday Functioning at Three-Year Follow-Up in Older Adults without Dementia. J. Alzheimers Dis..

[54] Tosun D., Mojabi P., Weiner M.W., Schuff N. (2010). Joint analysis of structural and perfusion MRI for cognitive assessment and classification of Alzheimer’s disease and normal aging. Neuroimage.

[55] Westerberg C., Mayes A., Florczak S.M., Chen Y., Creery J., Parrish T., Weintraub S., Mesulam M.M., Reber P.J., Paller K.A. (2013). Distinct medial temporal contributions to different forms of recognition in amnestic mild cognitive impairment and Alzheimer’s disease. Neuropsychologia.

[56] Wierenga C.E., Dev S.I., Shin D.D., Clark L.R., Bangen K.J., Jak A.J., Rissman R.A., Liu T.T., Salmon D.P., Bondi M.W. (2012). Effect of mild cognitive impairment and APOE genotype on resting cerebral blood flow and its association with cognition. J. Cereb. Blood Flow Metab..

[57] Xie L., Dolui S., Das S.R., Stockbower G.E., Daffner M., Rao H., Yushkevich P.A., Detre J.A., Wolk D.A. (2016). A brain stress test: Cerebral perfusion during memory encoding in mild cognitive impairment. Neuroimage Clin..

[58] Zou J.X., Wang M.J., Lei X.J., Chen X.G. (2014). 3.0 T MRI arterial spin labeling and magnetic resonance spectroscopy technology in the application of Alzheimer’s disease. Exp. Gerontol..

[59] McKhann G., Drachmann D., Folstein M., Katzmann R., Price D., Stadlan E. (1984). Clinical diagnosis of Alzheimer’s disease: Report of the NINCDS-ADRDA work group under the auspices of Department of Health and Human Services Task Force on Alzheimer’s Disease. Neurology.

[60] Jack C.R., Albert M.S., Knopman D.S., McKhann G.M., Sperling R.A., Carrillo M.C., Thies B., Phelps C.H. (2011). Introduction to the recommendations from the National Institute on Aging-Alzheimer’s Association workgroups on diagnostic guidelines for Alzheimer’s disease. Alzheimer’s Dement..

[61] Petersen R.C. (2004). Mild cognitive impairment as a diagnostic entity. J. Int. Med..

[62] Albert M.S., DeKosky S.T., Dickson D., Dubois B., Feldman H.H., Fox N.C., Gamst A., Holtzman D.M., Jagust W.J., Petersen R.C. (2011). The diagnosis of mild cognitive impairment due to Alzheimer’s disease: Recommendations from the national institute on aging-alzheimer’s association workgroups on diagnostic guidelines for Alzheimer’s disease. Alzheimer’s Dement..

[63] Jak A.J., Bondi M.W., Delano-Wood L., Wierenga C., Corey-Bloom J., Salmon D.P., Delis D.C. (2009). Quantification of five neuropsychological approaches to defining mild cognitive impairment. Am. J. Geriatr. Psychiatry.

[64] Winblad B., Palmer K., Kivipelto M., Jelic V., Fratiglioni L., Wa hlund L.O., Nordberg A., Backman L., Albert M., Almkvist O. (2004). Mild cognitive impairment–beyond controversies, towards a consensus: Report of the International Working Group on Mild Cognitive Impairment. J. Intern. Med..

[65] Dubois B., Feldman H.H., Jacova C., Cummings J.L., Dekosky S.T., Barberger-Gateau P., Delacourte A., Frisoni G., Fox N.C., Galasko D. (2010). Revising the definition of Alzheimer’s disease: A new lexicon. Lancet Neurol..

[66] Petersen R.C., Lopez O., Armstrong M.J., Getchius T.S.D., Ganguli M., Gloss D., Gronseth G.S., Marson D., Pringsheim T., Day G.S. (2018). Practice guideline update summary: Mild cognitive impairment: Report of the Guideline Development, Dissemination, and Implementation Subcommittee of the American Academy of Neurology. Neurology.

[67] Petersen R.C., Stevens J.C., Ganguli M., Tangalos E., Cummings J.L., DeKosky S.T. (2001). Practice parameter: Early detection of dementia: Mild cognitive impairment (an evidence-based review). Report of the Quality Standards Subcommittee of the American Academy of Neurology. Neurology.

[68] Petersen R.C., Aisen P.S., Beckett L.A., Donohue M.C., Gamst A.C., Harvey D.J., Jack C.R., Jagust W.J., Shaw L.M., Toga A.W. (2010). Alzheimer’s 985 Disease Neuroimaging Initiative (ADNI): Clinical characterization. Neurology.

[69] Musiek E.S., Chen Y., Korczykowski M., Saboury B., Martinez P.M., Reddin J.S., Alavi A., Kimberg D.Y., Wolk D.A., Julin P. (2012). Direct comparison of fluorodeoxyglucose positron emission tomography and arterial spin labeling magnetic resonance imaging in Alzheimer’s disease. Alzheimers Dement..

[70] Binnewijzend M.A., Benedictus M.R., Kuijer J., van der Flier W.M., Teunissen C.E., Prins N.D., Wattjes M.P., van Berckel B.N., Scheltens P., Barkhof F. (2016). Cerebral perfusion in the predementia stages of Alzheimer’s disease. Eur. Radiol..

[71] Sierra-Marcos A. (2017). Regional Cerebral Blood Flow in Mild Cognitive Impairment and Alzheimer’s Disease Measured with Arterial Spin Labeling Magnetic Resonance Imaging. Int. J. Alzheimers Dis..

[72] Toth P., Tarantini S., Csiszar A., Ungvari Z. (2017). Functional vascular contributions to cognitive impairment and dementia: Mechanisms and consequences of cerebral autoregulatory dysfunction, endothelial impairment, and neurovascular uncoupling in aging. Am. J. Physiol. Heart Circ. Physiol..

[73] Fleisher A.S., Podraza K.M., Bangen K.J., Taylor C., Sherzai A., Sidhar K., Liu T.T., Dale A.M., Buxton R.B. (2009). Cerebral perfusion and oxygenation differences in Alzheimer’s disease risk. Neurobiol. Aging.

[74] Tai L.M., Thomas R., Marottoli F.M., Koster K.P., Kanekiyo T., Morris A.W., Bu G. (2016). The role of APOE in cerebrovascular dysfunction. Acta Neuropathol..

[75] Bracko O., Cruz Hernández J.C., Park L., Nishimura N., Schaffer C.B. (2021). Causes and consequences of baseline cerebral blood flow reductions in Alzheimer’s disease. J. Cereb. Blood Flow Metab..

[76] Østergaard L., Engedal T.S., Moreton F., Hansen M.B., Wardlaw J.M., Dalkara T., Markus H.S., Muir K.W. (2016). Cerebral small vessel disease: Capillary pathways to stroke and cognitive decline. J. Cereb. Blood Flow Metab..

[77] Thomas K.R., Osuna J.R., Weigand A.J., Edmonds E.C., Clark A.L., Holmqvist S., Cota I.H., Wierenga C.E., Bondi M.W., Bangen K.J. (2021). Regional hyperperfusion in older adults with objectively-defined subtle cognitive decline. J. Cereb. Blood Flow Metab..

[78] Faber J., Fonseca L.M. (2014). How sample size influences research outcomes. Dent. Press J. Orthod..

